# Evaluation of the in vitro antibacterial activity of the solvent fractions of the leaves of *Rhamnus prinoides* L’Herit (Rhamnaceae) against pathogenic bacteria

**DOI:** 10.1186/s12906-016-1279-6

**Published:** 2016-08-15

**Authors:** Yalew Molla, Teshome Nedi, Getachew Tadesse, Haile Alemayehu, Workineh Shibeshi

**Affiliations:** 1 Department of Pharmacy, College of Health Sciences, Debre Markos University, P. O. Box, 269, Debre Markos, Ethiopia; 2 Department of Pharmacology and Clinical Pharmacy, School of Pharmacy, Addis Ababa University, P. O. Box, 1176, Addis Ababa, Ethiopia; 3 Department of Biomedical Sciences, College of Veterinary Medicine and Agriculture, Addis Ababa University, P. O. Box, 1176, Addis Ababa, Ethiopia; 4 Aklilu Lemma Institute of Pathobiology, Addis Ababa University, P. O. Box, 1176, Addis Ababa, Ethiopia

**Keywords:** Antibacterial activity, Minimum inhibitory concentration, *Staphylococcus aureus*, *Streptococcus pyogen*, *Streptococcus pneumoniae*, *Salmonella typhi*

## Abstract

**Background:**

Medicinal plants play great roles in the treatment of various infectious diseases. *Rhamnus prinoides* is one of the medicinal plants used traditionally for treatment of bacterial diseases. The antibacterial activity of the crude extract of the plant had been shown by a previous study, but this study was undertaken to further the claimed medicinal use of the plant by screening its solvent fractions for the said activity so that it could serve as a basis for subsequent studies.

**Methods:**

The solvent fractions of the plant were obtained by successive soxhlet extraction with solvents of increasing polarity, with chloroform and methanol, followed by maceration of the marc of methanol fraction with water. The antibacterial activity of the solvent fractions was evaluated on seven bacterial species using agar well diffusion method at different concentrations (78 mg/well, 39 mg/well and 19.5 mg/well) in the presence of positive and negative controls. The minimum inhibitory concentration of the solvent fractions was determined by micro-broth dilution method using resazurin as indicator.

**Result:**

Methanol and chloroform fractions revealed antibacterial activities against the growth of test bacterial strains with varying antibacterial spectrum and the susceptible bacterial species were *Staphylococcus aureus, Streptococcus pyogen, Streptococcus pneumoniae* and *Salmonella typhi*. The average minimum inhibitory concentration value of the methanol and chloroform fractions ranged from 8.13 mg/ml to 32.5 mg/ml and from 8.13 mg/ml to 16.25 mg/ml, respectively.

**Conclusion:**

The methanol and chloroform fractions demonstrated significant antibacterial activities against the growth of pathogenic bacteria but the aqueous fraction did not reveal antibacterial activity against any of the test bacteria.

## Background

Infectious diseases are the continuous major causes of death in the world, with a great impact in developing countries [1, 2]. The periodically emerging new and old infectious diseases greatly magnify the global burden of infectious diseases [3]. The majorities of emerging infectious events are caused by bacteria which can be associated with evolution of drug resistant strains and overwhelming of the natural host defenses [4, 5].

Therefore, the search of new or alternative mechanisms to effectively treat and prevent infectious diseases, particularly bacterial diseases, have to be encouraged to effectively reduce these global burden. Medicinal plants have been used for treatment of different infectious diseases and thus accepted as alternative approaches. In Africa, 90 % of the population relies on traditional healers to meet their primary healthcare needs [6] which indicate the presence of many medicinal plants with a potential source of new drugs.

*Rhamnus prinoides* L’Herit (Rhamnaceae) is one of the medicinal plants that have been used traditionally for the treatment of different infectious diseases. *Rhamnus prinoides* has different ethnomedicinal uses in different countries of Africa. In Kenya, traditionally, the different parts of plant are used in the management of Ear, Nose and Throat (ENT) infections, gonorrhea, malaria and brucellosis [7, 8]. In Ethiopia*,* the leaves, fruits or roots of *Rhamnus prinoides* is used to treat tonsillitis [9–13]. In addition, the different parts of the plant have been used in the management of scabies, hepatitis, tinea capitis, 'chiffea' (Eczema), ringworm and dandruff [11, 14–17]. Moreover, the leaf of the plant is used for the management of waterborne and related diseases [18].

The purpose of the study was to evaluate the in vitro antibacterial activities of the crude and solvent fractions of the leaves of *Rhamnus prinoides* against clinical and standard strains of selected bacterial species, which can in turn provide a clue for the isolation and identification of active principle(s) responsible for the antibacterial activity.

## Methods

### Collection and authentication of the plant material

The leaves of *Rhamnus prinoides* was collected from the cultivated garden of a family in October 2014, around Debre Markos Town, East Gojjam Zone, Amhara Region, 300 km away from Addis Ababa, North West Ethiopia and identified by a taxonomist at the National Herbarium, Department of Biology, College of Natural and Computational Sciences, Addis Ababa University, and the specimen (number YM002) was deposited for future references.

### Preparation of extracts

#### Crude extraction

One hundred fifty gram of coarsely powdered leaves was macerated in 80 % methanol for a period of 3 days with occasional shaking using a shaker (Bibby scientific limited stone Staffo Reshire, United Kingdom). The extract was filtered with Whatman No 1 filter paper (Schleicher and Schuell Microscience Gmbh, Germany). The residue was remacerated for the second and third times with fresh solvent. The extracted solution was combined and concentrated by Rota vapor (Buchi Rota-vapor R-200, Switzerland). Then, it was dried in a lyophilizer (Operan, Korea vacuum limited, Korea).

#### Solvent fractionation

The solvent fractionation was conducted by using both Soxhlet and maceration techniques. Soxhlet extraction was carried out by sequential extraction of the powdered leaves in solvents of increasing polarity viz. chloroform and methanol as described by a study [19]. The powdered leaf was extracted by chloroform at a temperature not exceeding 40 °C. This extraction process was continued exhaustively until clear solution in the timble was siphoned into the solvent flask. Then, the chloroform fraction was filtered with Whatman No. 1 filter paper and concentrated using rotary evaporator. The concentrated solution was dried in oven at a temperature of 40 °C.

The dried marc of the chloroform fraction was extracted using absolute methanol following the same procedure described for chloroform fraction. Finally, the dried marc of methanol fraction was macerated with distilled water and dried in the lyophilizer to get the aqueous fraction.

The percentage yield of the crude extract, aqueous fraction, methanol fraction, and chloroform fraction were 17.5 %, 4.32 %, 8.74 % and 6.21 %, respectively. Each dried crude extract and solvent fractions was reconstituted in sterile 20 % DMSO (for crude extract and the methanol fraction), 70 % DMSO (for the chloroform fraction) or sterilized distilled water (for aqueous fraction) to prepare the tested concentrations of the plant material.

#### Phytochemical screening

The qualitative phytochemical investigations of the crude extract, and chloroform, methanol and aqueous fractions of leaves of *Rhamnus prinoides* were carried out using standard tests [20–22]

#### Inoculum preparation and standardization

Standard and clinical isolates of different bacterial strains including *Escherichia coli (E. coli)* (American Type Culture Collection (ATCC) 25922), *E. coli* (clinical isolate), *Pseudomonas aeruginosa* (*P. aeruginosa)* (ATCC 27853), *P. aeruginosa* (Clinical isolate), *Staphylococcus aureus (S. aureus)* (ATCC 25923), *S. aureus* (clinical isolate), *Shigella flexneri (S. fleneri)* (ATCC 12022), *S. fleneri* (clinical isolates*)*, *Streptococcus pneumoniae (S.pneumoniae)* (ATCC 49619), *S. pneumoniae* (clinical isolate), *Streptococcus pyogen (S. pyogen)* (ATCC 19615)*, S.pyogen* (clinical isolate) and *Salmonella typhi (S. typhi)* (ATCC 13062) were obtained from Ethiopian Public Health Institution (EPHI). Then, each bacterial strain was inoculated and spread on pre-labeled nutrient agar and 5 % sheep blood agar (for streptococcus species) aseptically in a Safety Cabinet (Bioair instruments, Eurolone® Company, Italy) and incubated for 24 h at 37 °C. The bacterial turbidity of each bacterium was prepared by growth method and standardized by following the guideline of Clinical and Laboratory Standard Institute [23]. The turbidity of the inoculum tube was adjusted visually by either adding bacterial colonies or by adding sterile normal saline solution to that of the already prepared 0.5 McFarland standard.

### Antibacterial activity assay

#### Agar well diffusion

The antibacterial agar well diffusion assay was conducted by following the methods described previously [24]. The standardized bacterial broth culture prepared in section 2.4 were streaked evenly on sterile MHA plates or MHA with 5 % sheep blood (for streptococcus species) with cotton swab. After thirty minutes, on each plate, four equidistant wells were made with a 6 mm diameter sterilized cork borer. The labeled wells were filled with 100 μl of 780 mg/ml, 390 mg/ml, and 195 mg/ml of the crude or each of the solvent fractions making the final concentration of 78 mg/well, 39 mg/well and 19.5 mg/well respectively. In addition, the commercial antibiotic discs of ampicillin 0.01 mg/disc (for streptococcus species), cefoxitin 0.03 mg/disc (for *E. coli* and *S. aureus* species) and ciprofloxacin 0.005 mg/disc (for other bacterial strains) were used as a positive control. The solvents of the crude and each fraction were used as negative controls. Then, the plates were left undisturbed for about 2 h at room temperature. After incubation at 37 °C for 24 h, the zone of inhibition was measured using a ruler. The experiment was performed in three independent tests for each bacterial strains and the mean of zones of inhibition was calculated for each extract.

#### Determination of the Minimum Inhibitory Concentration (MIC)

The crude extract and solvent fractions that showed antibacterial activity by agar well diffusion method were subjected to serial resazurin based microtitre dilution technique on 96 well plates (Greiner Bio-One, Germany) as described by previous studies [25, 26]. The first column of microtiter plate was filled with 100 μl stock solution (390 mg/ml) of the crude or each active solvent fraction of test material except the last well in which equal amount of the respective solvent was added. Then, all the wells of microtitre plates were filled with sterilized 100 μl of MHB [23] or BHI (for streptococcus species) [27]. Two fold serial dilution of the crude extract or solvent fraction (throughout the row) was carried out until the 10th column. The 11th and 12th column were used as the growth control for labeled bacterium in which the 100 μl of the solvents of the extract or that of the solvent fraction was added. Then, 30 μl of 0.01 % w/v resazurin solution was added and mixed in each well.

Briefly, within 15 min of standardization to approximately 5 × 10^6^ CFU/mL with the respective broth, 20 μl of bacterial suspension was added to each well except 7th and 8th row which were reserved as extract color contrast control and sterility control, respectively. Subsequently, the serial dilution procedures gave rise to a final concentration of the plant material ranging from 130 mg/ml to 0.26 mg/ml.

Finally, after wrapping each plate loosely with parafilm, the plates were incubated at 37 °C for 24 h. In the experiment, each plate had a set of three controls: (a) a column with all solutions with the exception of the test extract or fraction, (b) a row with all solutions except bacterial suspension and the extract or fraction was used as sterility control, (c) a row with all solutions except the bacterial inoculums was used as color contrast control.

After incubation, any color change observed from purple to pink or colorless was taken as positive for growth of bacteria. The lowest concentration of plant leaf extract at which no color change occurred was recorded as the MIC value. All the experiments were performed in triplicates for each bacterium. The average value was taken for the MIC of test plant material.

#### Determination of Minimum Bactericidal Concentration (MBC)

MBC was determined by a method described in different studies [26]. In this technique, the contents of all wells containing a concentration of test material above the MIC value from each triplicate, in the MIC determination test, was streaked on MHA or MHA supplemented with 5 % sheep blood (for streptococcus species) with wire loop aseptically and incubated at 37 °C for 24 h. The lowest concentration of the extract which showed no bacterial growth after incubation was observed for each triplicate and noted as the MBC. The average value was taken for the MBC of test material against each bacterium.

#### Statistical analysis

The experimental data are expressed as mean ± Standard Error of the Mean (SEM). Data are analyzed using the Statistical Package for the Social Sciences (SPSS), version 16.0 software. The statistical differences of the mean zone of inhibition of crude extract and solvent fractions for individual bacterium was carried out by employing one way analysis of variance (ANOVA) followed by Tukey Post Hoc Multiple Comparison test at a significance level of *P* < 0.05.

## Results

### Antibacterial activity

The growth of test bacterial strains was inhibited by the tested concentrations of the crude extract, the methanol fraction and chloroform fraction of the leaves of *Rhamnus prinoides* in concentration dependent manner (Tables 1, 2 and 3). However, the aqueous fraction was devoid of an antibacterial activity against any of the test bacterium. Among the test bacteria, the most susceptible bacterium at 78 mg/well of crude extract was standard strains of *S. aureus* followed by clinical isolates of *S. aureus* and standard strains of *S. pyogen* with a mean zone of inhibition of 22 mm, 21.67 mm and 20.33 mm, respectively. In addition, among the gram negative bacteria, standard strains of *S. typhi* were most susceptible at 78 mg/well of the crude extract with the maximum mean zone of inhibition of 17.67 mm (Table 1).

**Table 1:** Zone of inhibition (in mm) of the different concentrations of crude extract and solvent fractions of the leaves of *Rhamnus prinoides* against gram positive bacteria

Category of test	Concentration	Bacteria					
		*S. aureus*		*S. pyogen*		*S. pneumoniae*	
		Stand	Clinic	Stand	Clinic	Stand	Clinic
Crude	19.5 mg/well	16.67 ± 0.88^a3d2^	16.67 ± 0.33^a3c1d3^	13.67 ± 0.88^a3c2d3^	12.33 ± 0.33^a3c1d2^	13.33 ± 0.33^a3d2^	11.67 ± 0.33^a3c2d3^
	39 mg/well	19.67 ± 0.88^a3^	19.00 ± 0.58^a3d1^	18.33 ± 0.67^a3^	16.33 ± 0.67^a3^	16.00 ± 1.00^a2^	15.33 ± 0.33^a3d2^
	78 mg/well	22.00 ± 0.58^a2^	21.67 ± 0.33^a2^	20.33 ± 0.33^a3^	18.67 ± 0.33^a3^	19.33 ± 1.20^a1^	18.00 ± 0.00^a3^
	Cef 0.03 mg/disc*	28.33 ± 0.58	26.33 ± 0.67	NT	NT	NT	NT
	Amp 0.01 mg/disc*	NT	NT	27.33 ± 0.67	26.33 ± 1.20	24.00 ± 0.58	23.00 ± 0.58
Methanol	19.5 mg/well	13.00 ± 0.58^a3d2e2^	11.67 ± 0.33 ^a3d2e3^	12.67 ± 0.33^a3d1^	11.67 ± 0.33^a3d2^	12.33 ± 0.88^a3d1^	11.33 ± 0.33^a3d3^
	39 mg/well	14.33 ± 0.33^a3f3^	13.33 ± 0.33^a3f3^	14.00 ± 0.58^a3f2^	12.67 ± 0.33^a3d1f3^	14.33 ± 0.33^a3^	13.67 ± 0.33^a3d1^
	78 mg/well	16.17 ± 0.44^a3g3^	15.33 ± 0.67^a3g3^	15.00 ± 0.58^a3g3^	15.00 ± 0.58^a3g3^	16.00 ± 0.58^a3^	15.33 ± 0.33^a3g2^
	Cef 0.03 mg/disc*	27.67 ± 0.33	26.33 ± 0 .33	NT	NT	NT	NT
	Amp 0.01 mg/disc*	NT	NT	28.00 ± 0.00	26.33 ± 0 .67	25.00 ± 0 .58	23.67 ± 0.33
Chloroform	19.5 mg/well	11.67 ± 0.33^a3d1e3^	11.67 ± 0.33^a3d1e3^	12.00 ± 0.58 ^a3d2^	10.67 ± 0.33^a3d2^	12.00 ± 0.58^a3d2^	11.00 ± 0.00^a3d2^
	39 mg/well	13.00 ± 0.58^a3f3^	13.33 ± 0.33^a3f3^	13.67 ± 0.33 ^a3f3^	12.00 ± 0.58 ^a3f3^	14.00 ± 0.58^a3^	13.00 ± 0.58^a3f2^
	78 mg/well	14.33 ± 0.33^a3g3^	14.00 ± 0.58^a3g3^	15.00 ± 0.58 ^a3g3^	13.67 ± 0.58^a3g3^	15.67 ± 0.33^a3g1^	15.00 ± 0.58^a3g3^
	Cef 0.03 mg/disc*	27.00 ± 0.57	26.33 ± 0.33	NT	NT	NT	NT
	Amp 0.01 mg/disc*	NT	NT	27.67 ± 0.33	27.00 ± 0.58	25.00 ± 0.58	23.67 ± 0.33

Values are expressed as Mean ± S.E.M (*n* = 3), analysis was performed with One-Way ANOVA followed by Tukey test; ^a^compared to positive control, ^b^to 19.5 mg/well, ^c^to 39 mg/well, ^d^to 78 mg/well, ^e^to crude 19.5 mg/well, ^f^to crude 39 mg/well, ^g^to crude 78 mg/well; ^1^ *P* < 0.05, ^2^ *P* < 0.01, ^3^ *P* < 0.001. The negative control has shown no antibacterial activity. * = positive controls, Stand = standard (ATCC) strains, Clinic. = clinically isolated strains, NT = not tested, Cef = Cefoxitin, Amp = ampicillin, Cip = Ciprofloxacin

**Table 2:** Zone of inhibition (in mm) of the different concentrations of crude and solvent fractions of the leaves of *Rhamnus prinoides* against gram negative bacteria

Category of test	Concentration	Bacteria			
		*S. flexneri*		*E. coli*	
		Stand	Clinic	Stand	clinic
Crude	19.5 mg/well	12.00 ± 0.58^a3d2^	11.67 ± 0.33 ^a3d2^	11.67 ± .88^a3d1^	11.33 ± 0.67^a3d2^
	39 mg/well	14.00 ± 0.00^a3d1^	13.67 ± 0.33 ^a3d1^	12.67 ± 0.33^a3^	12.67 ± 0.33^a3^
	78 mg/well	17.00 ± 0.58^a3^	16.00 ± 0.58 ^a3^	15.00 ± 0.00^a3^	14.50 ± 0.29 ^a3^
	Cef 0.03 mg/disc*	NT	NT	28.33 ± 0.88	27.00 ± 0.58
	Cip 0.005 mg/disc*	26.33 ± 0.67	25.00 ± 0.58	NT	NT
Methanol	19.5 mg/well	11.00 ± 0.00^a3d2^	10.67 ± 0.67^a3d2^	10.33 ± 0.33^a3d3^	9.00 ± 0.58^a3c2d3^
	39 mg/well	13.00 ± 0.58^a3^	12.67 ± 0.33^a3^	12.00 ± 0.58^a3d1^	11.67 ± 0.33^a3d2^
	78 mg/well	15.00 ± 0.58^a3^	14.67 ± 0.33^a3^	14.50 ± 0.29^a3^	14.33 ± 0.33^a3^
	Cef 0.03 mg/disc*	NT	NT	27.67 ± 0.33	26.67 ± 0.33
	Cip 0.005 mg/disc*	27.00 ± 0.58	26.33 ± 0.67	NT	NT
Chloroform	19.5 mg/well	10.00 ± 0.58^a3d2^	10.33 ± 0.33^a3^	---	---
	39 mg/well	11.33 ± 0.33^a3f1^	11.67 ± 0.67^a3^	----	---
	78 mg/well	13.33 ± 0.33 ^a3g2^	12.33 ± 0.33^a3g2^	---	---
	Cip 0.005 mg/disc*	25.00 ± 0.58	24.67 ± 0.33	NT	NT

Values are expressed as Mean ± S.E.M (*n* = 3), analysis was performed with One-Way ANOVA followed by Tukey test ^a^compared positive control, ^b^to 19.5 mg/well, ^c^to 39 mg/well, ^d^to 78 mg/well, ^e^to crude 19.5 mg/well, ^f^to crude 39 mg/well, ^g^to crude 78 mg/well; ^1^ *P* < 0.05, ^2^ *P* < 0.01, ^3^ *P* < 0.001. The negative control has shown no antibacterial activity. * = positive controls, Stand = standard (ATCC) strains whereas Clinic. = clinically isolated strains, NT = not tested, Cef = Cefoxitin, Cip = Ciprofloxacin, --- = no activitiy

**Table 3:** Zone of inhibition (in mm) of the different concentrations of crude and solvent fractions of the leaves of *Rhamnus prinoides* against gram negative bacteria

Category of test	Concentration	Bacteria		
		*P. aeruginosa*		*S. typhi*
		Stand	Clinic	stand
Crude	19.5 mg/well	11.00 ± 0.58^a3c1d2^	10.67 ± 0.88^a3^	13.33 ± 0.33^a3d2^
	39 mg/well	13.33 ± 0.33^a3^	13.00 ± 0.58^a3^	14.00 ± 0.58^a3d2^
	78 mg/well	14.33 ± 0.33 ^a3^	14.00 ± 0.58^a3^	17.67 ± 0.67^a3^
	Cip 0.005 mg/disc*	25.67 ± 0.33	24.33 ± 0.88	30.00 ± 0.58
Methanol	19.5 mg/well	10.33 ± 0.33^a3d1^	10.33 ± 0.33^a3c1d2^	11.00 ± 0.58^a3d2^
	39 mg/well	12.00 ± 1.00^a3^	12.33 ± 0.33^a3^	12.67 ± 0.88^a3d1^
	78 mg/well	13.67 ± 0.33^a3^	13.67 ± 0.33^a3^	15.67 ± 0.33^a3^
	Cip 0.005 mg/disc*	24.33 ± 0.33	24.00 ± 0.58	31.00 ± 0.58
Chloroform	19.5 mg/well	---	---	10.33 ± 0.33^a3d2e1^
	39 mg/well	---	---	11.67 ± 0.33^a3^
	78 mg/well	---	---	13.00 ± 0.58^a3g3^
	Cip 0.005 mg/disc*	NT	NT	30.33 ± 0.33

Values are expressed as Mean ± S.E.M (*n* = 3), analysis was performed with One-Way ANOVA followed by Tukey test; ^a^compared to positive control, ^b^to 19.5 mg/well, ^c^to 39 mg/well, ^d^to 78 mg/well, ^e^to crude 19.5 mg/well, ^f^to crude 39 mg/well, ^g^to crude 78 mg/well; ^1^ *P* < 0.05, ^2^ *P* < 0.01, ^3^ *P* < 0.001. The negative control has shown no antibacterial activity * = positive control, Stand = standard (ATCC) strains whereas Clinic = clinically isolated strains, Cip = Ciprofloxacin, --- = no activity

Similar to the crude extract, the most susceptible bacterium against methanol fraction was standard strains of *S. aureus* followed by standard strains of *S. pneumoniae* with a mean zone of inhibition of 16.17 mm and 16 mm, respectively, at 78 mg/well concentration. The zone of inhibition of methanol fraction at 78 mg/well against *S. typhi* was also greater than that of its activity against the other gram negative test bacterial strains (Tables 2 and 3).

The most sensitive bacterium against chloroform fraction at 78 mg/well concentration was standard strains of *S. pneumoniae* followed by standard strains of *S. pyogen* with a mean zone of inhibition of 15.67 mm, and 15 mm (Tables 1, 2 and 3). However, this fraction produced no antibacterial activity coverage against *P. aeruginosa* and *E. coli* regardless of their respective strains. Generally, the antibacterial activities of the chloroform fraction in terms of its zone of inhibition were slightly lower than that of the methanol fraction against each bacterial strain for which it was active, especially at 78 mg/well and 39 mg/well concentrations even though the difference at the corresponding concentration were not statistically significant.

### Minimum inhibitory concentration of crude extract and solvent fractions

As presented in Table 4, for the crude extract, the maximum MIC obtained was 8.13 mg/ml (against *E. coli, P. aeruginosa* and *S. pneumoniae* species) and the minimum MIC was 2.03 mg/ml (against staphylococcus species). Moreover, unlike the slight differences between the figures of zone of inhibition of the clinically isolated and standard strains within the same species, the MIC values were equal for the strains of the same species with the exception of *S. flexneri* for which the MIC of the clinical isolate is slightly higher (5.42 mg/ml) than that of its standard ones (4.06 mg/ml).

**Table 4:** The MIC (in mg/ml) of the crude extract and the solvent fractions of the leaves of *Rhamnus prinoides* against gram positive and gram negative bacteria

Bacteria		Crude extract	Solvent fractions	
			Methanol fraction	Chloroform fraction
		MIC	MIC	MIC
*S. aureus*	Stand.	2.03 ± 0.00	8.13 ± 0.00	8.13 ± 0.00
	Clinc.	2.03 ± 0.00	8.13 ± 0.00	16.25 ± 0.00
*S. pyogen*	Stand.	4.06 ± 0.00	8.13 ± 0.00	8.13 ± 0.00
	Clinc.	4.06 ± 0.00	8.13 ± 0.00	16.25 ± 0.00
*S. pneumoniae*	Stand.	8.13 ± 0.00	16.25 ± 0.00	16.25 ± 0.00
	Clinc.	8.13 ± 0.00	16.25 ± 0.00	16.25 ± 0.00
*S. flexneri*	Stand.	4.06 ± 0.00	16.25 ± 0.00	16.25 ± 0.00
	Clinc.	5.42 ± 1.36	16.25 ± 0.00	16.25 ± 0.00
*E. coli*	Stand.	8.13 ± 0.00	32.50 ± 0.00	---
	Clinc.	8.13 ± 0.00	32.50 ± 0.00	---
*P. aeruginosa*	Stand.	8.13 ± 0.00	16.25 ± 0.00	---
	Clinic	8.13 ± 0.00	16.25 ± 0.00	---
*S. typhi*	Stand	4.06 ± 0.00	16.25 ± 0.00	16.25 ± 0.00

MIC = Minimum Inhibitory Concentration, the values are the average of triplicate tests. Stand. = standard (ATCC) strains whereas Clinic. = clinically isolated strains

The MIC value for methanol fraction ranged from 32.5 mg/ml (against *E. coli* species) to 8.13 mg/ml (against *S. aureus* and *S. pyogen* species). The MIC figures of the chloroform fraction ranged from 8.13 mg/ml to 16.25 mg/ml in all bacterial species for which it was active. For the same bacterial species tested, the MIC of the two active solvent fractions were equal with the exception of clinically isolated strains of *S. aureus* and *S. pyogen* for which the methanol fraction was more potent than the chloroform fraction.

### Minimum bactericidal concentration of crude extract and solvent fractions

The MBC of the crude extract of the study plant was in the range of 16.25 mg/ml (against both strains of *S. pneumoniae*) to 2.03 mg/ml (against standard strains of Staphylococcus species). The corresponding values of the methanol fraction were 65 mg/ml (against clinical isolate of *E. coli*) and 13.54 mg/ml (against standard strain of *S. pyogen*). The MBC values of the chloroform fraction ranged from 32.5 mg/ml to 16.25 mg/ml against the growth of the susceptible bacterial species (Table 5). Taken together, the crude extract was more potent and killed the bacteria at lower concentration compared to that of the methanol and chloroform fractions.

**Table 5:** The MBC (in mg/ml) of the crude extract and the solvent fractions of *Rhamnus prinoides* against gram positive and gram negative bacteria

Bacteria		Crude extract	Solvent fractions	
			Methanol fraction	Chloroform fraction
		MBC	MBC	MBC
*S. aureus*	Stand.	2.03 ± 0.00	16.25 ± 0.00	16.25 ± 0.00
	Clinc.	4.06 ± 0.00	16.25 ± 0.00	16.25 ± 0.00
*S. pyogen*	Stand.	4.06 ± 0.00	13.54 ± 2.71	16.25 ± 0.00
	Clinc.	4.06 ± 0.00	16.25 ± 0.00	16.25 ± 0.00
*S. pneumoniae*	Stand.	8.13 ± 0.00	32.50 ± 0.00	32.50 ± 0.00
	Clinc.	16.25 ± 0.00	32.50 ± 0.00	32.50 ± 0.00
*S. flexneri*	Stand.	8.13 ± 0.00	32.50 ± 0.00	16.25 ± 0.00
	Clinc.	8.13 ± 0.00	32.50 ± 0.00	32.50 ± 0.00
*E. coli*	Stand.	8.13 ± 0.00	32.50 ± 0.00	---
	Clinc.	8.13 ± 0.00	65.00 ± 0.00	---
*P. aeruginosa*	Stand.	8.13 ± 0.00	32.50 ± 0.00	---
	Clinic.	8.13 ± 0.00	32.50 ± 0.00	---
*S. typhi*	Stand	4.06 ± 0.00	16.25 ± 0.00	16.25 ± 0.00

MBC = Minimum Bactericidal Concentration, the values are the average of triplicate tests. Stand. = standard (ATCC) strains whereas Clinic. = clinically isolated strains

### Phytochemical constituents of the crude extract and solvent fractions

According to the qualitative phytochemical screening study, the crude extract of the leaf of *Rhamnus prinoides* was found to be positive for the presence of all of the tested secondary metabolites except for cardiac glycosides, whereas the methanol fraction was positive for the presence of alkaloids, tannins, flavonoids, saponins, polyphenols and terpenoids. The chloroform fraction was confirmed for the presence of tannins, polyphenols, terpenoids, anthraquinones and flavonoids while the aqueous fraction contained only saponins (Table 6).

**Table 6:** Preliminary phytochemical investigation of the crude extract and the solvent fractions of leaves of *Rhamnus prinoides* using chemical test methods

Metabolites tested	Crude extract	Solvent fractions		
		Chloroform fraction	Methanol fraction	Aqueous fraction
Alkaloids	+	-	+	-
Saponins	+	-	+	+
Tannins	+	+	+	-
Anthraquinones	+	+	-	-
Polyphenols	+	+	+	-
Terpenoids	+	+	+	-
Flavonoids	+	+	+	-
Cardiac glycosides	-	-	-	-

+ = present, - = absent

## Discussion

The present study was undertaken to determine on which fractions do the constituents of the leaves of *Rhamnus prinoides* responsible for its antibacterial activity are concentrated. In addition, even though the antibacterial activity of the crude extract of the plant had been studied by a previous work in Gondar, Ethiopia [28], the crude extract was also evaluated in this study to assure its antibacterial activity as there was differences in geographical areas of plant collection, bacterial stains, solvent system and the parts of the plant used for extraction purpose.

According to the present study, the zones of inhibition of the bioactive fractions were lower than that of the crude extract at equal concentrations against the individual strains of test bacteria. The reason for the enhanced antibacterial activity of the crude extract might be due to the synergistic or additive effect of the secondary metabolites that were relatively partitioned in the solvent fractions as it had been extracted by the successive solvents of increasing polarity.

The crude extract and the methanol fraction had similar antibacterial activity profile in terms of antibacterial activity coverage and their activity was higher in the gram positive bacteria than that of the majority of gram negative test bacteria at their comparable concentration. Similarly, the mean zone of inhibition of chloroform fraction at its comparable concentration against gram positive bacteria was also greater than that of gram negative bacteria. Obviously, it is not surprising that gram negative bacteria were less susceptible for the crude and the active solvent fractions of the study plant as they have an outer membrane which precludes the partial penetration of the bioactive phytochemicals unlike that of the gram positive bacteria having less effective permeability barrier [29, 30].

Unlike methanol fraction, the chloroform fraction was not active against the clinical and standard strains of *P. aeruginosa* and *E. coli*. In addition, these species were the least susceptible bacteria for the methanol fraction and crude extract at equal concentrations compared to the other test bacteria. The possible reason for the less susceptible nature of these bacterial species could be due to the differences in the resistance mechanisms to the bioactive compounds detected in each solvent fraction among the test bacteria. For example, *P. aeruginosa* and *E. cloi* have the inherent ability of producing different resistance mechanisms like efflux pump [31], expressing AmpC β-lactamase [32] or biofilm formation [33] which could hinder the antibacterial activity of the bioactive compounds detected in the chloroform fraction.

Furthermore, the differences in the antibacterial spectrum of the methanol and the chloroform fraction against the test bacteria might be linked to the differences in the composition and/or the concentration of the secondary metabolites in the respective fractions. For instance, unlike the chloroform fraction, the presence of alkaloids and saponins in methanol fraction could directly or indirectly enhance its bacterial growth inhibitory effects on gram negative bacteria including *E. coli* and *P. aeruginosa* which were not responsive to the chloroform fraction [34].

The zones of inhibition of the methanol fraction were slightly higher than that of chloroform fraction against each of susceptible strains for which both were active, at equal concentrations. This may be associated with the less diffusible components of the chloroform fraction on the aqueous surfaces of the agar plate [35, 36] and their differences in the composition and concentrations of bioactive secondary metabolites.

Unlike the methanol and chloroform fractions, the aqueous fraction was found to be devoid of antibacterial activities against all the test bacterial strains regardless of the concentrations tested. Such type of result in the present study is concordant with other similar study following the same solvent fractionation principles in which the aqueous fraction was not having antibacterial activities [37]. The plausible reason for this phenomenon might be due to the localization of the secondary metabolites of the plant in the chloroform and methanol fractions as the plant had been extracted in sequential solvents of increasing polarity. The localization of bioactive metabolites in the active fractions can be supported by the fact that most of the secondary metabolites of the medicinal plants have aromatic rings and hence could be extracted by the organic solvents used sufficiently before being extracted by water [38], which can further be strengthened by the absence of almost all of the secondary metabolites tested in aqueous fraction.

The antibacterial screening findings in terms of zone of inhibition of the crude and the active solvent fractions of *Rhamnus prinoides* against the respective susceptible bacteria were inversely proportional to their values of MIC and MBC, with some exception like for *S. pneumoniae*, that is, the more susceptible the bacteria to the crude extract or the solvent fraction, the less is its corresponding MIC and MBC values, suggesting the reproducibility and consistency of the experiments. In addition, in almost all findings, the MBC value was equals to or one dilution factor greater than that of the MIC value for each bacterium in the microbroth dilution tests of crude extract and the active solvent fractions, which might indicate the sensitivity of the dilution method in detecting the minimum bacterial turbidity that indicated the growth of bacteria than that of the visual inspection method in which ambiguity of determining the MIC value is common [39].

However, the MIC values of clinical isolate of *S. flexneri* for the crude extract and that of clinical isolate of *S. aureus* and *S. pyogen* for the chloroform fraction were greater than the corresponding values of the standard strains of the same species. In addition, the MBC values of clinical isolates of *S. aureus* and *S. pneumoniae* for crude extract; *S. pyogen* and *E. coli* for methanol fraction*;* and *S. flexneri* for chloroform were greater than the corresponding values of the standard strains of the same species. These differences in the potency of the crude extract and the active solvent fractions against the strains of the same bacterial species might be associated to the susceptibility differences between the strains in which the clinical isolates could have a higher chance of developing a resistance mechanism of decreasing the access of the bioactive metabolites to the target sites since they had been isolated from the clinic settings in which resistant strains are common [40].

Groups of phytochemical compounds commonly associated antimicrobial activity in medicinal plants are flavonoids, alkaloids, tannins, triterpenoids, essential oils, saponins, glycosides and phenols [41, 42]. Therefore, the antibacterial activity of the leaves of *Rhamnus prinoides* might be attributed to either the individual class of these compounds present in the crude and the active solvent fractions or to the synergistic effect that each class of compound exerted to give the observed antibacterial activity findings.

## Conclusion

The present study revealed that the chloroform fraction and the methanol fraction of the leaves of *Rhamnus prinoides* have antibacterial activities against the growth of the selected pathogenic bacteria with varying antibacterial spectrum. Therefore, this study provides scientific support for the traditional use of the medicinal plant in the treatment of bacterial infections that are probably caused by the susceptible bacteria. Further studies aimed at the isolation and identification of active substance in the crude extract, methanol and chloroform fractions of the leaves of *Rhamnus prinoides* could disclose compounds with better therapeutic values.

## Abbreviations

DMSO: Dimethylsulfoxide
ENT: Ear, nose and throat
*S. aureus*: Staphylococcus aureus
ATCC: American type cell collection
MIC: Minimum inhibitory concentration
MBC: Minimum bactericidal concentration
MHA: Mueller hinton agar
MHB: Mueller hinton broth
*S. pyogen* species: Staphylococcus pyogen
